# Evidence of cardiac electrical remodeling in patients with Huntington disease

**DOI:** 10.1002/brb3.1077

**Published:** 2018-07-20

**Authors:** Ksenija Cankar, Ziva Melik, Jan Kobal, Vito Starc

**Affiliations:** ^1^ Faculty of medicine Institute of Physiology University of Ljubljana Ljubljana Slovenia; ^2^ Division of Neurology University Medical Centre Ljubljana Ljubljana Slovenia

**Keywords:** advanced ECG analysis, autonomic nervous system, clinical indices for heart disease, Huntington's disease, QT variability

## Abstract

**Objective:**

Although Huntington's disease (HD) is a disease of the central nervous system, HD mortality surveys indicate heart disease as a major cause of death. Cardiac dysfunction in HD might be a primary consequence of peripherally expressed mutant huntingtin or secondary to either a general decline in health or the onset of neurological dysfunction. The aim of the study was to clarify the heart muscle involvement.

**Materials and Methods:**

We measured conventional and advanced resting ECG indices. Thirty‐one subjects with a confirmed huntingtin gene mutation and 31 age‐ and gender‐matched controls were included. The HD subjects were divided into four groups based on their Unified Huntington Disease Rating Scale (UHDRS) motor score.

**Results:**

We detected changes in advanced ECG variables connected with electrical ventricular remodeling (*t* test, *p* < 0.01). The increase in the unexplained part of both QT variability and the standard deviation of normal‐to‐normal QT intervals, presumably reflecting beat‐to‐beat changes in repolarization, was most pronounced. Further, both variables correlated with the product of the cytosine–adenine–guanine (CAG) triplets’ repeat length and the subjects’ age (CAP), the former *R* = 0.423 (*p* = 0.018) and the latter *R* = 0.499 (*p* = 0.004). There was no correlation between the CAP score and any of variables representing autonomic nervous system activity.

**Conclusions:**

Both autonomic nervous system dysfunction and cardiac electrical remodeling are present in patients with HD. The changes in advanced ECG variables observed in the study evolve with HD progression. The increased values of QT unexplained variability may be a marker of temporal inhomogeneity in ventricular repolarization associated with malignant ventricular arrhythmias.

## 1 INTRODUCTION

Huntington's disease (HD) is a hereditary, progressive, neurodegenerative disorder caused by a mutation in the Huntingtin gene (IT15 gene, located on the short arm of chromosome 4), which codes for the protein Huntingtin. Huntingtin gene in patients with HD contains abnormally long sequence of three DNA bases—cytosine–adenine–guanine (CAG). Although it is a disease of the central nervous system, mortality surveys indicate that heart disease is one of the major causes of death in patients with HD (Sørensen & Fenger, 1992). However, the mechanisms of cardiac pathophysiology in patients with HD remain unknown. It is not fully understood whether cardiac dysfunction is a primary consequence of peripherally expressed mutant huntingtin (Cui et al., 2006) or secondary to either the general decline in health or the onset of neurological dysfunction (Abildtrup & Shattock, 2013).

Studies investigating heart involvement in patients with HD have focused mostly on the determination of altered autonomic nervous system (ANS) activity via assessment of heart rate variability (HRV), specifically demonstrated as an inverse correlation between the severity of clinical HD symptoms as assessed by the Unified Huntington Disease Rating Scale (UHDRS) and the modulation of the cardiovagal activity (Andrich et al., 2002). Increased sympathetic activity has also been observed in presymptomatic HD mutation carriers (Kobal et al., 2010). However, to our knowledge, no study with HD patients has been performed in which the intrinsic properties of the heart were assessed, such as those related to the electrical remodeling of the heart as observed by different patterns in depolarization and repolarization spread directions.

In the past 20 years, multiple advanced resting ECG techniques have improved the diagnostic or prognostic value of ECG in detecting human cardiac diseases, even before the onset of clinical signs or changes in the conventional ECG (Schlegel et al., 2010). Besides the measurement of HRV, which has been used predominantly for the assessment of sympathovagal balance, these techniques include indices of the electrical remodeling of the ventricles (e.g., the QRS‐T angle [Kardys et al., 2003]), ventricular depolarization (e.g., high‐frequency QRS [HFQRS] ECG [Abboud, Berenfeld, & Sadeh, 1991; Schlegel et al., 2004]), and ventricular repolarization. The indices of ventricular repolarization comprise various indices of QT interval variability (QTV), such as the QT variability index (QTVI) (Atiga et al., 1998; Berger et al., 1997; Starc & Schlegel, 2006) and the unexplained part of QTV (unexplained QTV), which represents an intrinsic part of QTV independent of simultaneous heart rate variability, the influence of the heart rate on QTV having been eliminated (Starc & Schlegel, 2008).

For example, in a study by Kardys et al. (2003), an increased spatial QRS‐T angle had more prognostic value for fatal endpoints than any classic cardiovascular risk factor, including diabetes. Finally, a number of studies show that the index of QT interval variability QTVI (Berger et al., 1997) can identify patients at risk for sudden cardiac death (Atiga et al., 1998; Haigney et al., 2004; Piccirillo et al., 2007), possibly even more than both left ventricular ejection fraction (as assessed by ultracardiography) and T‐wave alternans (Atiga et al., 1998; Berger et al., 1997).

At present, advanced ECG analysis seems to be the best noninvasive method to detect the heart condition of a patient. Hence, we speculated that advanced resting ECG indices, such as those representing electrical remodeling of the ventricles, QTV indices, and HFQRS indices, might be a sensitive tool to detect heart disease, particularly in the later stages of HD.

## 2 MATERIAL AND METHODS

### 2.1 Subjects

In this study, a total of 33 HD subjects with confirmed huntingtin gene mutations were included. There were no juvenile patients with HD and no patients who had been over sixty years at the disease onset enrolled in this study. Gene mutation was determined by way of the extended number of CAG triplets on the huntingtin gene. Subjects were divided into four groups according to their UHDRS motor score (Huntington Study Group, 1996) and groups cutoffs according to Andrich et al. (2002): presymptomatic HD (PHD) subjects, score 0–4; early symptomatic HD patients (EHD), score 6–23; midsymptomatic HD patients (MHD), score 25–41; and late symptomatic HD patients (LHD), score 53–88. Two of the enrolled patients with HD were later excluded due to a left bundle branch block observed on an ECG recording. For each individual HD subject, a gender‐ and age‐matched healthy control was assigned. Basic details of the HD subject groups and control groups are reported in Table 1. There was no statistically significant difference in BMI or in the number of CAG triplets among groups of HD subjects.

**Table 1:** Basic details of the Huntington's disease (HD) subject groups and control groups

	PHD	PHD controls	EHD	EHD controls	MHD	MHD controls	LHD	LHD controls
Age	41.8 ± 6.5	40.8 ± 7.3	44.3 ± 12.0	45.0 ± 10.0	55.7 ± 9.4	56.5 ± 8.5	49.8 ± 11.3	50.8 ± 11.9
Gender	3M 3F	3M 3F	3M 6F	3M 6F	3M 3F	3M 3F	6M 4F	6M 4F
BMI	23.1 ± 2.5	24.5 ± 2.1	24.6 ± 4.1	25.0 ± 3.6	24.4 ± 2.8	22.8 ± 3.0	25.2 ± 3.4	24.5 ± 2.5
CAG	43.0 ± 4.2	—	44.3 ± 3.6	—	44.7 ± 3.2	—	46.9 ± 7.6	—
UHDRS	1.2 ± 1.8	—	12.1 ± 6.0	—	31.8 ± 6.9	—	70.1 ± 13.0	—

Note

M: males; F: females.

The study was approved by the national ethics committee, and informed consent was signed by each subject or his/her legal guardian.

### 2.2 Methods

ECG measurements were performed by the personal computer‐based ECG system Cardiax (IMED Co Ltd, Budapest, Hungary) with a frequency response to 300 Hz and a sampling rate of 1,000 samples/s. CardioSoft Enhanced Cardiology software (Houston, USA) was utilized to acquire a minimum of 256 waveforms acceptable for both signal averaging and variability analyses and used to acquire simultaneously the 12‐lead conventional and advanced ECG data as outlined below.

### 2.3 Conventional ECG variables

Signals from the conventional ECG were analyzed automatically by CardioSoft software. The RR, PR, QRS, QT/QTc, and JT/JTc intervals (Bazett‐corrected) as well as the frontal plane QRS‐ and T‐wave axes, P‐, QRS‐, and T‐wave amplitudes, and ST‐segment levels were determined.

Adhering to the interpretative criteria set by Corrado and McKenna (Corrado & McKenna, 2007), conventional 12‐lead ECGs were defined as being “abnormal” when any of the following criteria were present: (a) left atrial enlargement; (b) ST‐segment depression in 2 or more leads; (c) pathological Q waves; (d) inverted T waves in >2 consecutive leads; (e) left axis deviation/left anterior hemiblock; (f) right axis deviation/left posterior hemiblock; (g) prolonged QTc interval; or (h) Brugada‐like (coved type) early repolarization. According to these criteria, two of the enrolled HD subjects were excluded from further analysis due to the presence of a left bundle branch block (Berger et al., 1997).

### 2.4 Advanced ECG variables derived from signal averaging and connected with electrical ventricular remodeling

Signal averaging to acquire P‐, QRS‐, and T‐wave signal‐averaged templates for each channel was performed using custom software developed by the authors at the University of Ljubljana and at NASA's Johnson Space Center (Schlegel et al., 2010; Starc & Schlegel, 2006) to generate results for the variables of three‐dimensional ECG.

The vectorcardiogram was derived using the Frank‐lead reconstruction technique of Kors and coworkers (Kors, van Herpen, Sittig, & Bemmel, 1990) to establish several vectorcardiographic variables as previously described by Draper and coworkers (Draper, Peffer, Stallmann, Littmann, & Pipberger, 1964), including the spatial mean QRS‐T angle (Kardys et al., 2003). In this study, QRS‐T angles were determined between vectors at the peak value of the QRS complex and T‐wave (QRS‐T peak) and between the corresponding mean QRS and T‐wave vectors (QRS‐T mean).

### 2.5 Advanced ECG variables derived from variability analyses

Time series (~256 beats) for the RR and QT intervals were analyzed according to recommendations by the Task Force of the European Society of Cardiology and the North American Society of Pacing and Clinical Electrophysiology. Specific variability analyses included:

1. the standard deviation of normal‐to‐normal RR and QT intervals (SDNN_RR and SDNN_QT in all ECG leads, respectively);
2. several other time and frequency domain indices of RR interval variability, including the very low (0.0–0.04 Hz), low (0.04–0.15 Hz), high (0.15–0.40 Hz), and total (0.0–0.40 Hz) frequency powers of RR interval variability in natural log‐transformed units (ln ms2/Hz) calculated using autoregression (lnAR) and the Lomb periodogram method (lnLo) (Schlegel et al., 2010);
3. the QT variability index (QTVI) (Atiga et al., 1998), using the means and variances of the RR interval (Piccirillo et al., 2007) rather than those of the heart rate (Berger et al., 1997) in the denominator of the QTVI equation; and
4. the “unexplained” part of QTV (Solaimanzadeh et al., 2008; Starc & Schlegel, 2008), wherein the QTV signal is decomposed into two parts, one being described by the concomitant RR interval HRV and/or by the concomitant variability of the QRS‐T angle and the other representing the “unexplained” part of QTV. Decomposition is performed according to a model (Solaimanzadeh et al., 2008; Starc & Schlegel, 2008) that takes into account the hysteresis‐like properties of QT interval dynamics (Lang, Flapan, & Nielsen, 2001) as also the fact that while changes in QT intervals are predominantly driven by changes in RR intervals (Almeida et al., 2006), they can also occur in response to changes in QT wavefront direction descriptors, such as in the QRS‐T angle or equivalent (Acar, Yi, Hnatkova, & Malik, 1999; Kors, van Herpen, & Bemmel, 1999). In a specific manner, we determined the “unexplained” part of SDNN_QT (unexplained SDNN_QT) and the corresponding “unexplained” part of QTV (unexplained QTV).

### 2.6 Statistical analysis

The Sigmaplot software version 14.0 (Systat Software, USA) was used for statistical analysis and graphs construction. The mean values of all obtained standard and advanced ECG indices were calculated. All results obtained were compared among four groups of HD subjects by way of analysis of variance or, when the normality test or equal variance failed, the Kruskal–Wallis one‐way analysis of variance on ranks. Dunnett’s post hoc test was used. A comparison of each HD subject group with its control group was conducted using the Student *t* test or, when the normality test or equal variance failed, the Mann–Whitney rank sum test. In addition, the evaluation of the impact of the number of CAG triplets and the stage was assessed by considering the continuous variable disease burden. For this reason, a CAG age product (CAP) was calculated from the CAG repeat length and the current age of the subject: CAP = 100 × AGE × [(CAG – *L*) ÷ *S*]. *L* and *S* are constants; the values *L* = 30 and *S* = 627 were used according to Ross and coworkers (Ross et al., 2014). The CAP score provides an index of the length and severity of the individual's exposure to the effects of the mutant *HTT* gene, which is useful for conveying longitudinal data from cohorts of patients with a range of ages and CAG repeat lengths. All variables were compared to the CAP score; plots were calculated for the purpose of linear regression (Pearson correlation coefficient).

All results are expressed as mean values and standard deviation of the means or as medians and percentiles, with the level of significance at *p* < 0.05.

## 3 RESULTS

### 3.1 Conventional ECG variables

The representative conventional ECG indices values in all‐HD‐subjects groups and all control groups are presented in Table 2. Statistically significantly lower mean values of the QT interval (Dunnett's test, *p* = 0.002), the mean RR interval (*p* = 0.005), the SDNN (*p* = 0.004), and the RMSSD of the RR interval (*p* = 0.006) were observed in the HD patient group (including EHD, MHD, and LHD) compared with values obtained in the age‐ and gender‐matched control group. In addition, there was significantly lower power in the LF band (lnLoLF *p* = 0.04 and lnARLF *p* = 0.003) of the patients with HD and lower power in the HF band (lnLoHF *p* = 0.007 and lnARHF *p* = 0.002) of the HD patients compared with their controls. There was no statistically significant difference among the four groups of HD subjects and also no difference in any of the conventional ECG variables among four groups of the healthy controls.

**Table 2:** Conventional ECG parameters in Huntington's disease (HD) subjects and their controls

	PHD	PHD controls	EHD	EHD controls	MHD	MHD controls	LHD	LHD controls
RR mean	1,000 ± 136	1,014 ± 119	861 ± 126	1,007 ± 132	885 ± 264	1,036 ± 125	898 ± 98	978 ± 142
SDNN RR	54.5 ± 31.3	52.8 ± 21.6	30.1 ± 13.1	52.9 ± 21.0	38.7 ± 24.2	53.3 ± 19.5	33.7 ± 14.7	45.3 ± 18.7
RMSSD RR	36.4 ± 20.4	37.3 ± 17.2	23.7 ± 16.5	41.2 ± 18.8	26.6 ± 21.8	39.6 ± 23.0	20.2 ± 12.9	36.7 ± 22.1
QT mean	403.3 ± 30.1	415.7 ± 19.6	383.1 ± 21.0	416.1 ± 24.9	400 ± 45.2	430.3 ± 31.7	396.9 ± 16.1	415.7 ± 35.3
QTc	404.5 ± 18.0	411.5 ± 16.3	417.8 ± 20.6	415.0 ± 23.1	429.8 ± 23.3	428.8 ± 36.7	421.0 ± 15.0	427.3 ± 33.9
QRS axis	29.7 ± 44.9	47.0 ± 28.0	39.8 ± 38.6	50.3 ± 23.7	30.2 ± 14.0	42.5 ± 34.1	41.9 ± 19.7	47.0 ± 26.8
lnLoHF	4.88 ± 1.4	5.17 ± 0.6	4.23 ± 1.5	5.16 ± 1.0	4.19 ± 2.2	5.18 ± 1.4	3.48 ± 1.32	4.82 ± 1.2
lnLoLF	5.26 ± 1.0	5.26 ± 0.8	4.56 ± 0.7	5.49 ± 0.9	5.03 ± 1.3	5.29 ± 0.9	4.65 ± 1.2	5.06 ± 0.9
lnARHF	5.90 ± 1.3	6.46 ± 0.7	5.21 ± 1.7	6.38 ± 1.0	5.11 ± 2.3	6.35 ± 1.4	4.64 ± 1.4	6.17 ± 1.4
lnARLF	6.31 ± 1.0	6.60 ± 0.9	5.51 ± 0.9	6.82 ± 1.0	5.93 ± 1.4	6.71 ± 0.9	5.69 ± 1.3	6.40 ± 1.0

No correlations between the conventional ECG indices values and the CAP score were observed in the HD subjects.

### 3.2 Advanced ECG variables derived from signal averaging

Statistically significant differences in the advanced ECG variables between the all‐HD‐subjects group and their gender‐ and age‐matched controls are presented in Table 3.

**Table 3:** Advanced ECG parameters in Huntington's disease (HD) subjects and their controls

	HD subjects	Controls	*p*‐Values
QRST peak	51.8 ± 29.7	32.0 ± 21.7	0.0047
QRST mean	56.6 ± 27.7	41.0 ± 24.2	0.025
QTVI	−1.45 ± 0.46	−1.81 ± 0.36	0.0015
Unexplained QTV	−1.33 ± 0.87	−1.96 ± 0.68	0.0028
SDNN_QT	2.13 ± 0.77	1.89 ± 0.79	0.263
Unexplained SDNN_QT	1.66 ± 0.98	1.13 ± 0.63	0.017

**Figure 1:**
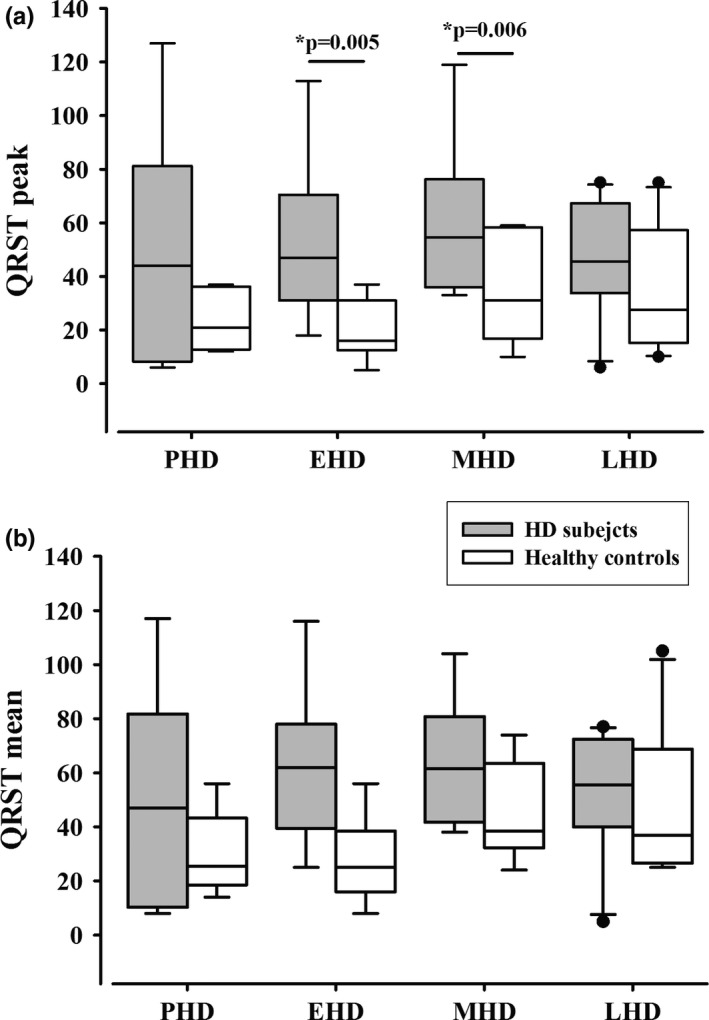
(a) QRS‐T angle peak values and (b) QRS‐T angle median values (25th; 75th percentile) in PHD subjects, EHD, MHD, and LHD patients and their control groups. (*statistically significant difference between EHD and MHD patients and their controls)

Increased QRS‐T peak values were observed in the EHD and MHD patients compared with their controls (*t* test, *p* < 0.05; Figure 1). In contrast, there was no difference in the QRS‐T mean values between the HD subjects groups and their controls. There was also no statistically significant difference among the four groups of HD subjects or among the four groups of healthy controls.

### 3.3 Advanced ECG variables derived from variability analysis

The all‐HD‐subjects group had significantly higher QTVI, unexplained QTV, SDNN_QT, and unexplained SDNN_QT values compared to the controls (Table 3).

The greatest differences between the HD and the control groups were observed among the corresponding unexplained part of QTV: unexplained QTV and unexplained SDNN_QT. Standard QTV variables containing both the explained and unexplained part, such as QTVI, also exhibited differences.

**Figure 2:**
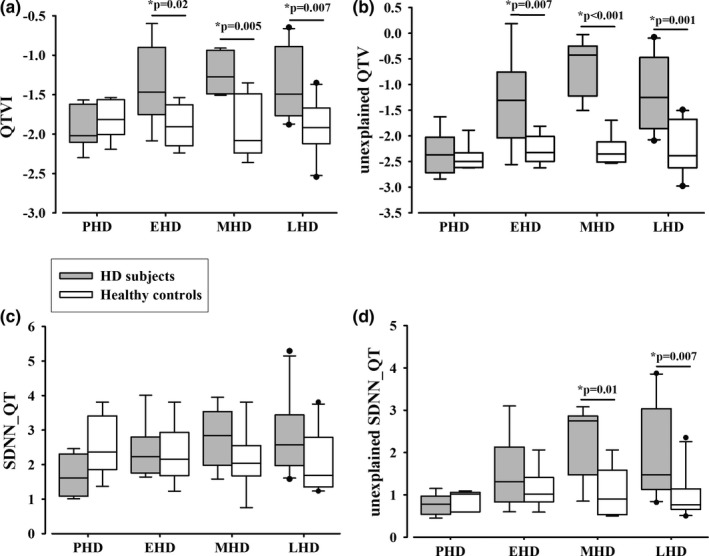
(a) QTVI, (b) unexplained QTV, (c) SDNN_QT, and (d) unexplained SDNN_QT median values (25th; 75th percentile) in PHD subjects, EHD, MHD, and LHD patients and their control groups. (*statistically significant difference between EHD, MHD, and LHD patients and their controls)

No differences in QTVI and unexplained QTV values were observed between the asymptomatic PHD subjects and their healthy age‐ and gender‐matched controls. In contrast, increased QTVI and unexplained QTV values were present in the EHD, MHD, and LHD patients compared with their controls (ANOVA, Dunnett's test, *p* < 0.01; Figure 2).

There were no statistically significant differences in SDNN_QT values between the HD subject groups and their control groups; however, unexplained SDNN_QT values were significantly higher in MHD and LHD patients compared with their controls (Dunnett's test, *p* < 0.01).

There were significantly less negative QTVI and unexplained QTV values in the EHD, MHD, and LHD groups compared with the PHD group of subjects (Dunnett's test, *p* < 0.05). There was also significantly higher unexplained SDNN_QT values in the MHD and LHD groups compared with the PHD group (Dunnett's test, *p* < 0.05). In contrast, there was no statistically significant difference between the four groups of healthy controls.

**Figure 3:**
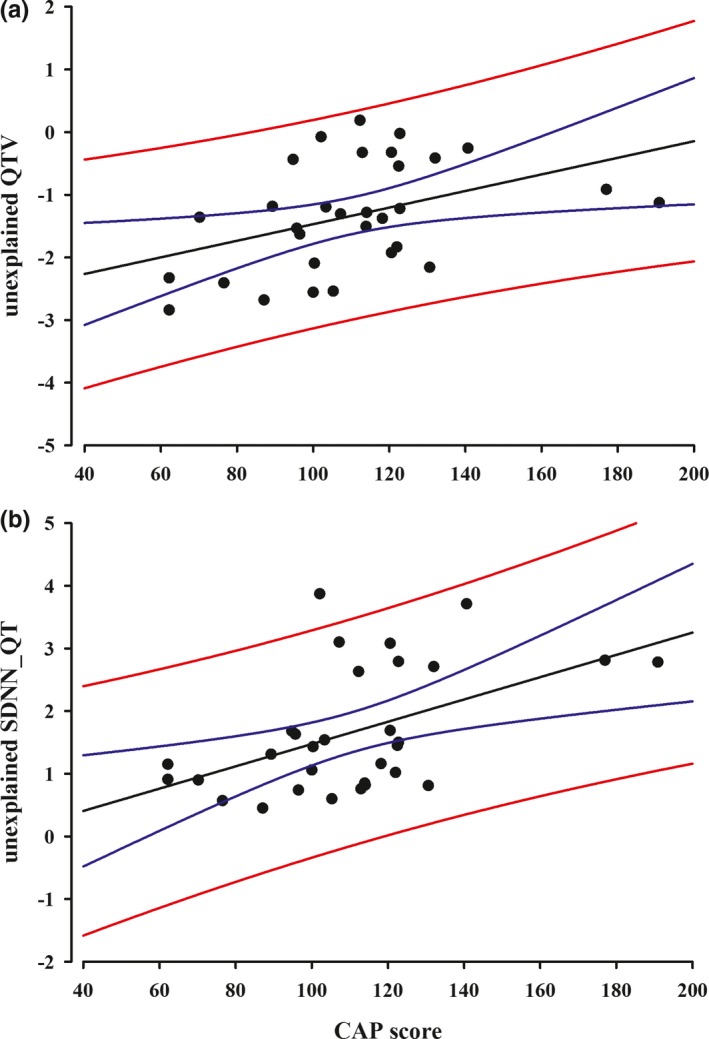
(a) Correlation between unexplained QTV values and CAP score (*N* = 31), (b) correlation between unexplained SDNN_QT and CAP score (*N* = 31)

Unexplained QTV values (Figure 3a) and unexplained SDNN_QT (Figure 3b) positively correlated with the CAP score (*p* = 0.017, *R* = 0.423 and *p* = 0.004, *R* = 0.499). In contrast, there was no statistically significant correlation between the CAP score and any of variables representing ANS activity.

## 4 DISCUSSION

The main findings of the present study are differences in advanced ECG variables between HD subjects and their age‐ and gender‐matched controls. The most pronounced changes in the variables related to electrical ventricular remodeling (QRS‐T peak, QRS‐T mean) as well as augmentation of QTVI and especially its unexplained part are observed in patients with HD in all stages of the disease except PHD.

There are only a limited number of studies that have investigated heart involvement in patients with HD, and the results of these epidemiological studies are controversial (Abildtrup & Shattock, 2013; Sørensen & Fenger, 1992). A great problem is the inaccuracy of death certificates. A frequently reported cause of death in the past was simply Huntington disease (Haines & Conneally, 1986). In the latest study, Rodrigues and coworkers reported the most frequent cause of death “other” and the second most frequent “unknown” (Rodrigues et al., 2017). This is probably due to the fact that these patients very often die in nursing homes, where there may be no precise determination of the cause of death. Some studies indicate that heart disease is one of the major causes of death in patients with HD (Abildtrup & Shattock, 2013; Lanska, Lavine, Lanska, & Schoenberg, 1988; Haines & Conneally, 1986; Sørensen & Fenger, 1992), although in age‐ and sex‐matched controls the same is true (Lanska, Lavine, Lanska, & Schoenberg, 1988).

Although HD is essentially a neurodegenerative disease, a mutant huntingtin aggregation in the nucleus and mitochondria of cardiomyocytes was observed (Mihm et al., 2007), so it might be worth investigating possible cardiac pathology in this disease. To our knowledge, the relevant data at present are almost exclusively from animal models of HD. There are no ultrasonographic data about heart function in patients with HD. The reason might be the difficulty of monitoring because of uncontrolled movements in the advanced stages of the disease.

We found only some differences in the standard ECG variables of RR and QT interval duration and in the HRV variables, when comparing patients to their controls. However, numerous studies indicate that conventional ECG variables may not be the most reliable predictors of heart failure (Acar, Yi, Hnatkova, & Malik, 1999; Kardys et al., 2003; Man et al., 2012; Bacharova & Estes, 2017; Schlegel et al., 2010).

The obtained differences in the HRV variables between patients with HD and their controls are in accordance with previous clinical studies, suggesting that a profound ANS dysfunction often accompanies HD (Andrich et al., 2002). The potential cardiac consequences and possible mechanisms of ANS dysfunction in patients with HD have, however, received little attention. HRV has been shown to be altered in HD subjects (Andrich et al., 2002; Kobal et al., 2010). An inverse correlation between the severity of clinical HD symptoms assessed by UHDRS and the modulation of cardiovagal activity has been found (Andrich et al., 2002). Disrupted circadian rhythms can alter central autonomic pathways, such as blood pressure changes and loss in the circadian control of heart rate. Nocturnal dipping is part of this normal circadian pattern, and its absence is associated with more severe end‐organ damage and increased risk of cardiovascular events. Recently, Bellosta Diago and coworkers published a study demonstrating PHD and EHD subjects to be more likely nondippers than their healthy controls (Bellosta Diago et al., 2018). Nocturnal dipping is a phenomenon describing a normal blood pressure decrease during sleep. An absence of nocturnal dipping seems to be a consequence of abnormal functioning of the ANS in HD. In our previous study, an attenuated sympathetic ANS response during mental stress and an augmented sympathetic ANS response to a cold pressure test in PHD and EHD subjects were demonstrated (Kobal et al., 2010). In contrast, no difference in HRV during orthostasis, the Valsalva maneuver, or a deep breathing test was noticed. Such a pattern of response modulation might be mediated through changed activity in cortical and subcortical structures involved in high‐order autonomic control. In addition, in the present study, a decrease in parasympathetic and sympathetic activity represented as tone fluctuation was also observed in the patients with EHD, MHD, and LHD. According to Schwartz and coworkers, sympathetic activation can trigger malignant arrhythmias, whereas vagal activity may exert a protective effect (Schwartz, La Rovere, & Vanoli, 1992). The ANS dysfunction observed in our study might be explained by the insular cortex atrophy present in patients with HD (Douaud et al., 2006).

A decrease in the standard ECG variables of RR and QT interval duration, representing changes in sympathetic nervous system activity, was observed in HD patients compared with their controls. These results are in accordance with another set of clinical studies that suggest a deregulated sympathetic nervous system, which plays a major role in the pathophysiology of congestive heart failure and has an impact on the morbidity and mortality of congestive heart failure patients (Esler & Kaye, 2000), such also having been reported in patients with HD (Sharma et al., 1999).

ANS dysfunction has additionally been reported in several animal transgenic lines (Kiriazis et al., 2012). An elevation of plasma noradrenaline level in association with reduced myocardial content and a greater number of active neurons in the brain regions that are known to regulate ANS activity were also observed in this animal model.

In addition to the assessed alterations of sympathovagal balance, pronounced differences in advanced ECG variables were observed between HD subjects and their controls. In the present study, a constant increase in QTVI (Berger et al., 1997; Starc & Schlegel, 2006) and unexplained QTV (the unexplained part of QTV that represents an intrinsic part of QTV independent of simultaneous heart rate variability)(Starc & Schlegel, 2008) was observed in the EHD, MHD, and LHD patients compared to their controls. Both QTVI and unexplained QTV are markers of temporal inhomogeneity in ventricular repolarization associated with malignant ventricular arrhythmias and are strong predictors of sudden cardiac death (Solaimanzadeh et al., 2008; Piccirillo et al., 2007). Studies utilizing the unexplained part of QTV are rare and require a deterministic model of QT variability (Starc & Schlegel, 2008) that enables separation of the explained and unexplained part of QTV. In one such study, increased IUQTV (index of unexplained QTV in lead II, defined as the ratio of the unexplained and explained part of QTV) and decreased SD2 (second standard deviation) on the Poincaré plot were most predictive of mortality in patients with familial dysautonomia compared to age‐ and gender‐matched healthy controls, suggesting that ventricular depolarization might be a cause of death (Solaimanzadeh et al., 2008).

The increase in unexplained QTV indicates the existence of alterations in cardiac muscle independent of ANS dysfunction. These alterations are greatest in the patients with LHD, a finding that is in accordance with the mitochondrial apoptosis present only at the end stage of the disease (Kuljis et al., 2012). Due to the observed lower T‐wave amplitude in the patients with LHD and their age‐matched control subjects, we also applied the T‐wave amplitude correction of the SDNN_QT and QTVI indices as well as the corresponding unexplained indices (Schmidt, Baumert, Malberg, & Zaunseder, 2016), but this did not provide any additional information.

The reported augmentation in ventricular repolarization variability in HD patients is consistent with the progressive cardiac dysfunction reported in the transgenic mouse models of HD. Mutant huntingtin aggregation in the nucleus and mitochondria of cardiomyocytes has been found to be associated with alteration in the mitochondrial ultrastructure and myocardial atrophy (Mihm et al., 2007; Kiriazis et al., 2012). However, the mechanisms of cardiac failure remain largely unstudied in both clinical settings and experimental animal models of HD.

Furthermore, electrical remodeling of the heart observed as changes in the overall magnitude and direction of the heart's electrical depolarization and repolarization and manifested as an altered QRS‐T angle (Kardys et al., 2003) was detected in the HD subjects, suggesting it as another possible marker of increased risk of cardiac mortality in patients with HD.

A key limitation of the present study is its small sample size, which is related to the fact that the population of HD subjects is relatively small compared to other neurodegenerative disorders. Despite this, statistically significant differences between the HD subject group as a whole and their age‐ and gender‐matched controls were observed in some standard and in most of the important advanced ECG variables. Observed differences evolved with disease progression. It would be desirable to collect advanced ECG data during some provocation tests (e.g., orthostasis, mental stress test, deep breathing) and to compare obtained results with resting measurements. Unfortunately, provocation testing is not possible to perform with MHD and LHD patients. In the future, it would be valuable to compare the advanced ECG variables with the mechanical properties of cardiac muscle.

## 5 CONCLUSIONS

Our results show that both ANS dysfunction and cardiac electrical remodeling are present in HD subjects as recorded by the ECG. Changes in the advanced ECG variables observed in this study evolve with HD progression. They are also regarded as independent predictors of sudden cardiac death in different subject populations and in different diseases (Piccirillo et al., 2007; Yamazaki et al., 2005). For this reason, the evaluation of these advanced ECG variables might also be a predictor of the risk of cardiac arrhythmia in patients with HD.

## CONFLICT OF INTEREST

The authors declare no conflict of interests.
